# Internal displacement and the Syrian crisis: an analysis of trends from 2011–2014

**DOI:** 10.1186/s13031-015-0060-7

**Published:** 2015-10-01

**Authors:** Shannon Doocy, Emily Lyles, Tefera D. Delbiso, Courtland W. Robinson, The IOCC/GOPA Study Team

**Affiliations:** Center for Refugee and Disaster Response, Johns Hopkins Bloomberg School of Public Health, 615 N. Wolfe Street, Baltimore, MD 21205 USA; Center for Research and Epidemiology in Disasters, Catholic University of Louvain, Clos Chapelle-aux-Champs, Bte B1.30.15, 1200 Brussels, Belgium

**Keywords:** Internal displacement, Syria, Conflict, Health

## Abstract

**Background:**

Since the start of the Syrian crisis in 2011, civil unrest and armed conflict in the country have resulted in a rapidly increasing number of people displaced both within and outside of Syria. Those displaced face immense challenges in meeting their basic needs. This study sought to characterize internal displacement in Syria, including trends in both time and place, and to provide insights on the association between displacement and selected measures of household well-being and humanitarian needs.

**Methods:**

This study presents findings from two complementary methods: a desk review of displaced population estimates and movements and a needs assessment of 3930 Syrian households affected by the crisis. The first method, a desk review of displaced population estimates and movements, provides a retrospective analysis of national trends in displacement from March 2011 through June 2014. The second method, analysis of findings from a 2014 needs assessment by displacement status, provides insight into the displaced population and the association between displacement and humanitarian needs.

**Results:**

Findings indicate that while displacement often corresponds to conflict levels, such trends were not uniformly observed in governorate-level analysis. Governorate level IDP estimates do not provide information on a scale detailed enough to adequately plan humanitarian assistance. Furthermore, such estimates are often influenced by obstructed access to certain areas, unsubstantiated reports, and substantial discrepancies in reporting. Secondary displacement is not consistently reported across sources nor are additional details about displacement, including whether displaced individuals originated within the current governorate or outside of the governorate. More than half (56.4 %) of households reported being displaced more than once, with a majority displaced for more than one year (73.3 %). Some differences between displaced and non-displaced population were observed in residence crowding, food consumption, health access, and education.

**Conclusions:**

Differences in reported living conditions and key health, nutrition, and education indicators between displaced and non-displaced populations indicate a need to better understand migration trends in order to inform planning and provision of live saving humanitarian assistance.

## Background

Since the beginning of the Syrian crisis in 2011, uprising and civil unrest have resulted in widespread displacement both within and outside of Syria. Population movements are linked to violence with the largest displaced populations coming from governorates where the greatest violence has occurred. Displacement is a survival strategy of endangered and deprived populations but there have also been instances of deliberate and forced displacement by both the Government and opposition forces [1] and indications that all parties are using displacement as a tool for demographic change to create geographic areas with more homogenous populations [2]. The Syrian Government has been judged as failing in its obligations to protect its population from and during forced displacement [3].

Following a dramatic increase in 2013, the number of internally displaced populations (IDPs) has continued to grow with many IDPs having moved multiple times because a single move has not protected them as battle lines constantly change and a breakdown of basic services spreads across the country. In addition to the massive scale of internal displacement, the flow of refugees into neighboring countries is also substantial and threatens to escalate tensions elsewhere in the region. The United Nations Refugee Agency (UNHCR) registered more than 3.2 million Syrian refugees as of October 2014, with Lebanon, Turkey, and Jordan, respectively, hosting the largest refugee populations [4].

Indications are that conflict, displacement, and the humanitarian crisis in Syria will persist, where large areas of the country are controlled by rebel groups, and possibly escalate in the near future as the international coalition continues airstrikes against the Islamic State (IS). The inability to accurately assess the status, size, and location of affected populations in Syria hampers humanitarian planning and provision of life saving assistance [5]. The aim of this study is to characterize internal displacement in Syria, including trends in both time and place, and to provide insights on the association between displacement and selected measures of household well-being and humanitarian needs.

## Methods

This paper presents findings from two complementary methods that provide different types of evidence on displacement in Syria. The first method, a desk review of displaced population estimates and movements, provides a retrospective analysis of national trends in displacement from March 2011 through June 2014. The second method, analysis of findings from a 2014 needs assessment by displacement status, provides insight into the displaced population and the association between displacement and humanitarian needs. A more detailed description of the needs assessment methodology and general findings is presented in Doocy et al., [6].

### Desk review

The desk review sought to determine the number of IDPs by governorate monthly and characterize displacement trends over the course of the conflict. The scope was limited to publically available websites including international organizations and United Nations agencies, organizations involved in the humanitarian response, donors, and other sources including academic institutions, think tanks, advocacy groups, and news organizations. Peer reviewed journal publications were not included in the desk review because it was anticipated that few, if any, studies with primary data focusing on internal displacement in Syria would be identified; furthermore, the time delay associated with peer review publication would render any existing studies outdated for the purposes of estimating current displacement. Publications from January 2011 and after were included which includes a several month period prior to the start of the conflict in March 2011. All identified sources were evaluated as potential sources of information for (i) estimation of IDP figures, locations and flows, and (ii) development of a situational timeframe that could inform development of IDP estimates and their progression over time. The key information sources identified with regular reporting are presented in Table 1; a total of 159 documents from eight sources (i.e., eight publication types from five different organizations) were included in addition to 15 other references that were not part of routine reporting.

**Table 1:** Overview of information sources with routine IDP reporting

Organization	Title	Update frequency	# of documents from source	Comments
ACAPS	Syria Needs Assessment Project (SNAP) Regional Assessments [31]	Approximately Monthly	19	Specific reporting of IDP figures by numerous sources. Often provides a breakdown of IDPs by governorate. Appears to be the most in-depth report with diverse reliable sources
OCHA	Humanitarian Snapshot: Syrian Arab Republic [7]	Intermittent	7	Map identifying size of population in need per governorate and indicating population movement
OCHA	Humanitarian Bulletin: Syrian Arab Republic [8]	Approximately Bi-Weekly	45	UN estimates of IDPs; sometimes includes discussion of IDP trends
OCHA	Humanitarian Dashboard [9]	Intermittent	12	UN estimates of IDPs
UNICEF	Syria Crisis Bi-Weekly Humanitarian Situation Report [6]	Approximately Bi-Weekly	41	Fairly consistent reporting on affected population size; IDP numbers not always identifiable
ECHO	ECHO Factsheet Syria [32]	Approximately monthly	21	Number of IDPs from Syrian Ministry of Local Administration [MoLA], SARC, OCHA
ECHO	ECHO Humanitarian Implementation Plan: Syria Crisis [33]	Intermittent	10	Reports number of IDPs, but includes no discussion of displacement trends
SARC	Syrian Arab Red Crescent Bulletin [5]	Approximately monthly	4	Occasionally reports total numbers of IDPs
Total Number of Documents Included			159	

Detailed information on IDP estimates and locations was extracted, including type(s) of information included in the document, breakdown of information by geographic governorate, data collection and/or reporting time frame, and data source type. Governorate level monthly IDP estimates were developed by reviewing available data and assessing source quality and credibility. When multiple estimates for a given location and time were available, the estimate assessed to be the most robust was identified and an explanatory note for the decision was provided. Sources were evaluated for quality and credibility based upon the sponsoring organization (i.e., government, international organization local organization), clear description of methodology, length of time between publication and estimation of estimates, and frequency of reporting. Sources were considered robust if they offered regular reporting of IDPs including source and/or methodology specification and disaggregated data available (gender, age, geographic location, etc.). Where there was more than one credible estimate, a range of values or average of multiple values was provided. When there were no [credible] estimates, imputed values from proximate and/or similar areas or modeled estimates were used (displacement were rates derived from the number of total IDPs at that period and applied population estimates for the particular governorate and time period). The final estimates of IDPs by governorate and year/month were presented with a central, or mid-range, estimate, as well as with low-range and high-range estimates, consistent with standard demographic estimation practice.

### Needs assessment

**Fig. 1:**
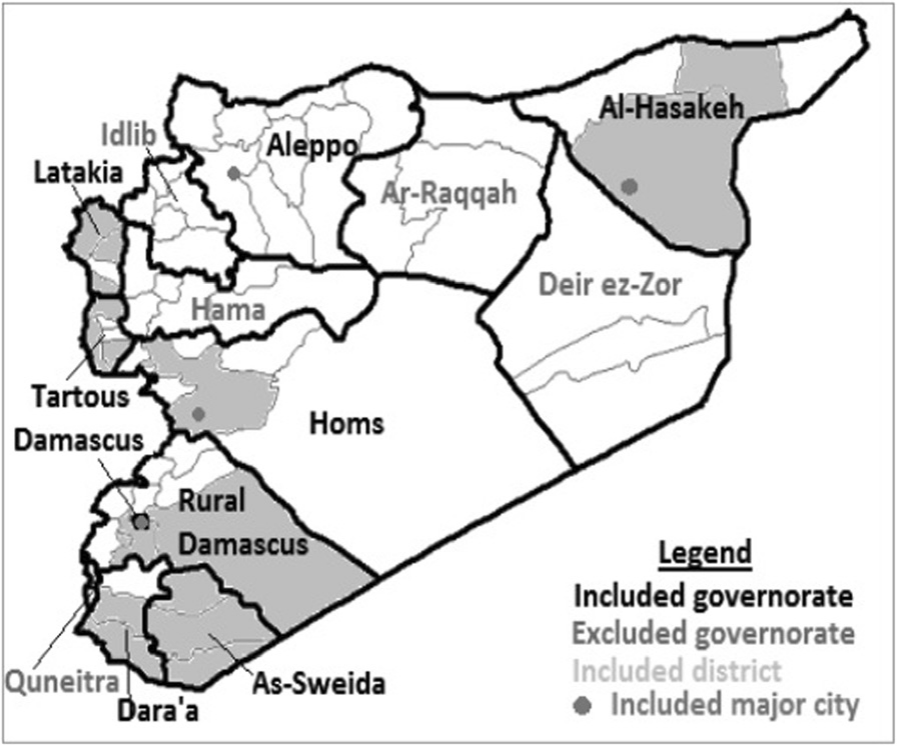
Areas included in the needs assessment

Between April and June 2014, International Orthodox Christian Charities (IOCC), an International non-governmental organization (NGO), and the Greek Orthodox Patriarchate of Antioch and All the East (GOPA) conducted a needs assessment of 3869 Syrian households affected by the crisis with the objective of gaining a better understanding humanitarian needs and assistance priorities. Given that no recent and accurate nationwide estimates of the displaced population or the population in need of assistance were available, planning a representative sample was exceptionally difficult; furthermore, security and access issues limited the ability to attain the desired geographic coverage. The assessment included 36 neighborhoods in 19 districts (of a total of 65 districts) in nine governorates (of a total of 14 governorates) (Fig. 1). Included neighborhoods met the following criteria: 1) no recent needs assessment from other organizations was available; and 2) large numbers of displaced or otherwise conflict-affected families perceived as vulnerable, poor or underserved with humanitarian aid were present [per the judgment of IOCC/GOPA staff implementing assistance programs]. Neighborhoods were excluded if they met any of the following criteria: 1) the assessment could present a security threat to interviewers or respondents; 2) significant humanitarian assistance was being received; or 3) the neighborhood was perceived as affluent [per the judgement of IOCC/GOPA staff; there were no explicit criteria used, assessment was subjective].

The assessment was intended to sample different types of households in the community; in each location, the planned sample was 30 households (in many cases multiple locations within a community were sampled) Religion was not a consideration in community or household selection. Eligible households included those that were displaced; host families of those displaced; returnees; and those otherwise directly affected by the conflict (including those with damaged or destroyed homes, conflict related deaths or disabilities or household members with special needs). For areas with large numbers of families registered with IOCC/GOPA, including those receiving and not receiving humanitarian assistance, a list-based sampling approach was used where households were randomly selected using interval sampling. For areas with few or no registered households, local community leaders were asked to refer the survey team to underserved families; where possible, multiple sources of referral were sought in each community. Lists were combined and cross-checked to prioritize families listed multiple times; interval sampling was then used to identify the remaining sample of households from the list.

Data was collected using a structured multi-sectoral paper questionnaire developed by IOCC/GOPA based on information needs to inform humanitarian assistance programming. The questionnaire was adapted from Sphere assessments, piloted in Syria, then revised based on experiences during the pilot, IOCC/GOPA program staff that conducted the interviews had prior experience with both humanitarian programming and data collection, including needs assessment and program monitoring information. Interviewers received training on household selection, interviewing techniques, and the survey tool, and were provided with a survey guide. Potential respondents were informed that participation was voluntary and that no humanitarian assistance or other direct benefit would result from participation in the survey. Verbal consent was sought, and if obtained the interview, which typically lasted 20–30 min, was carried out. Data was entered in Google Forms, exported into excel for cleaning and coding, and subsequently imported into SPSS 19 and EpiInfo for analysis. Sample weights were derived from the most recent available information on the population with unmet needs and were used to ensure the representativeness of the survey result to the governorates [4]. Pearson’s chi-square test of association was used to assess the association between displacement status and indicators included in the survey. Permission to conduct the assessment was sought from community leaders prior to approaching households for interviews; local ethical review approval was not required because the primary aim of the assessment was not research. The Johns Hopkins School of Public Health Institutional Review Board (IRB) approved the analysis of the de-identified data set as non-human subjects research.

## Results

### Source appraisal

A comprehensive review of information on displacement in Syria identified the Assessment Capacities Project (ACAPS) Regional Analysis for Syria and its predecessor, the Disaster Needs Analysis, as the most in-depth, regularly updated, and methodologically sound sources for governorate-level IDP estimates. The ACAPS Regional Analysis for Syria, developed by The Syria Needs Analysis Project (SNAP), provides a monthly compilation of published and unpublished information on the humanitarian situation in Syria beginning in January 2013. Its predecessor, the Disaster Needs Analysis, reports information on the unrest leading to conflict in Syria from December 2011 through December 2012. Each report presents an overview of governorate-level conflict, displacement, operational constraints, and sector information. Displacement figures and movement data are drawn by ACAPS from reliable sources including the United Nations Office for the Coordination of Humanitarian Affairs (OCHA) Humanitarian Bulletins, the Syrian Ministry of Local Administration (MoLA), Syrian Arab Red Crescent (SARC), and the Joint Rapid Assessments (J-RANS) conducted by the Assessment Working Group for Northern Syria. The majority of the sources identified in the desk review were those cited by ACAPS. Due to irregular reporting since the start of the crisis (Syrian Arab Red Crescent Bulletin [7]), regular reporting of overall affected population figures with sporadic IDP estimates (UNICEF’s Syria Crisis Bi-Weekly Humanitarian Situation Report [8]), or cross-citation between documents (OCHA documents [9–11]), figures reported by ACAPS were determined to be the most consistently reliable. Overall, given the high levels of conflict and insecurity, the limited humanitarian access, and the highly dynamic rates and volume of internal (and external) displacement, it is not surprising that IDP estimates are somewhat imprecise and do not present a highly nuanced picture at the aggregate level. That said, the ACAPS analysis, both in its Disaster Needs Analysis and the later SNAP, provides a consistent source of robust analysis of the various primary sources of data on internal and external displacement over several years.

A number of additional sources were identified that, while not intended to provide regular updates on displacement, offer a more comprehensive and nuanced portrait of the context of IDP estimation and internal displacement itself throughout the country. The most robust of these sources were two thematic reports released after the desk review’s June 2014 cutoff; one a compilation of Syrian governorate profiles compiled by OCHA and the other an analysis of internal displacement performed by the Internal Displacement Monitoring Center (IDMC) [12, 13].

### Estimates and trends in internal displacement

**Fig. 2:**
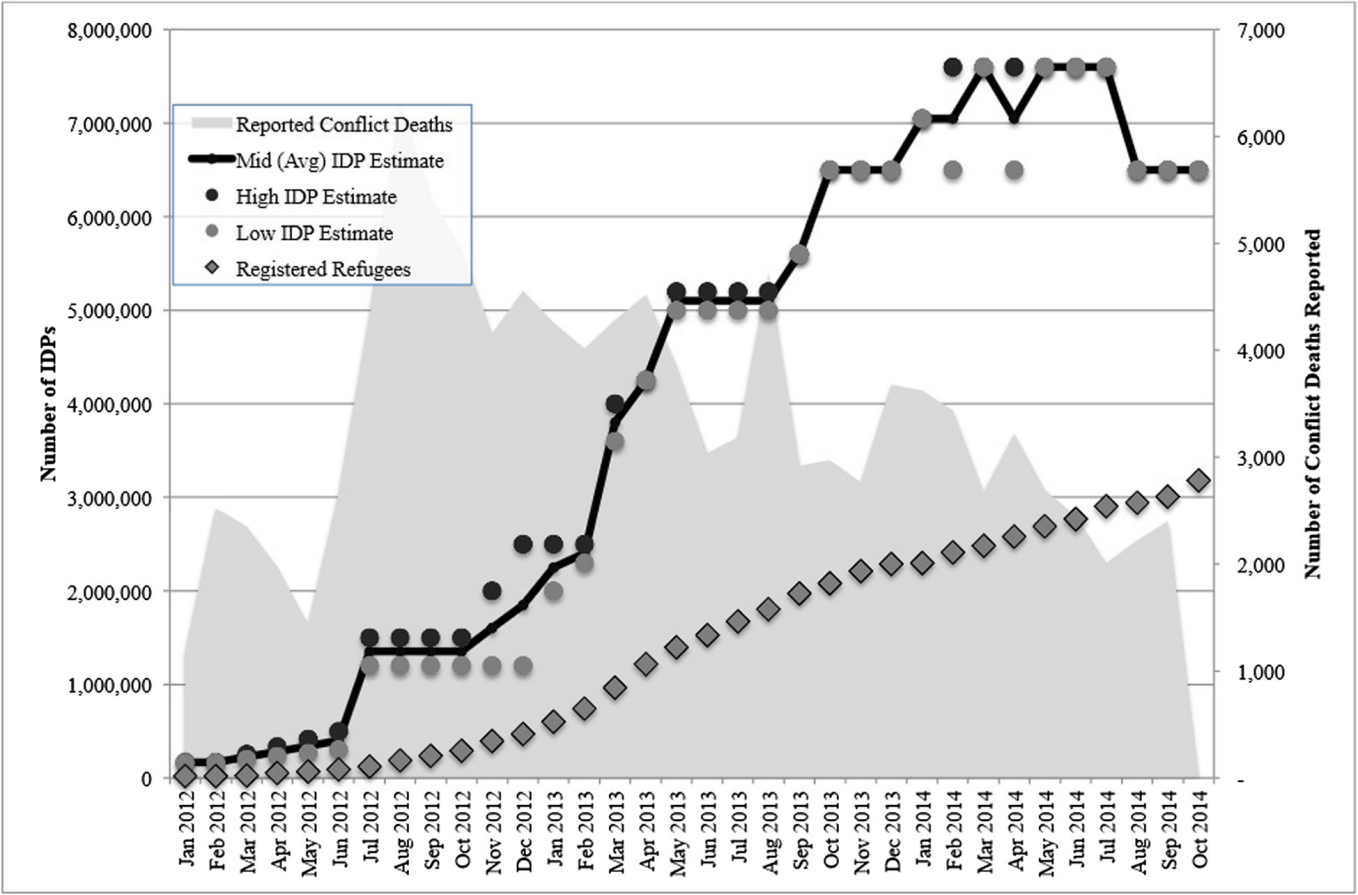
Estimated number of conflict-affected IDPs in Syria: January 2012–October 2014

Estimates of conflict-affected IDPs are based primarily on ACAPS analysis, which in turn derives from collation and analysis of various primary sources of data on internal displacement, including OCHA Humanitarian Bulletins, the MoLA, SARC, and J-RANS. There are periods of several months—including July-November 2012, May-August 2013, October-December 2013, and January-October 2014. Figure 2 presents trends in the total number of IDPs in Syria over time with high and low monthly IDP estimates largely from ACAPS analysis, both for the sake of consistency and also because ACAPS provides the most robust and transparent approach of the available sources. By October 2013, ACAPS was reporting only a single estimate of 6.5 million IDPs, without ranges or other sub-breakdowns. Following the release of the Syria Integrated Needs Assessment (SINA) in December 2013, the February 2014 ACAPS Regional Analysis Syria reported an updated estimate of 7.6 million IDPs. ACAPS sites this figure as the highest and, by their estimation, the most accurate between OCHA and SINA IDP estimates [14]. In October 2014 however, ACAPS returned to the previous estimate of 6.5 million IDPs citing only the estimate provided in OCHA’s August 2014 governorate profiles report [12]. No updated displacement estimates have been reported since August 2014.

**Fig. 3:**
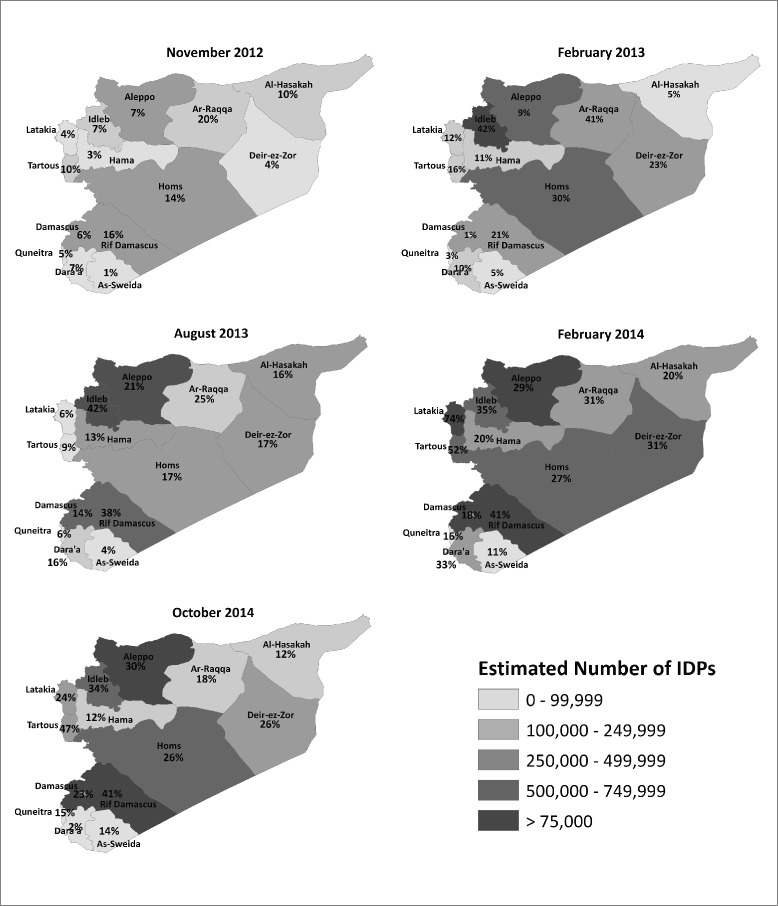
Geographic distribution of internal displacement over time

Maps of IDP populations are also presented at the governorate level to provide insight into geographic patterns of displacement and change over time. Displacement, using national estimates available for different time periods (November 2012 [15], February 2013 [16], August 2013 [17], February 2014 [14], October 2014 [18]) is shown in Fig. 3 to illustrate geographic shifts in the displaced population over the course of the conflict. Displacement is presented as both the absolute numbers of IDPs within each governorate and also expressed as a proportion of the 2011 pre-conflict population in each governorate [19]. The most recently reported absolute number of IDPs by governorate is presented in Table 2.

**Table 2:** Number of IDPs by governorate, October 2014

Governorate	Number of IDPs
Al-Hasakeh	197,500
Aleppo	1,787,000
Ar-Raqqa	177,000
As-Sweida	69,000
Damascus	410,600
Dara’a	26,600
Deir ez-Zor	441,000
Hama	245,500
Homs	560,000
Idleb	708,000
Latakia	300,000
Quneitra	72,000
Rif Damascus	770,000
Tartous	452,000

Reported displacement in the Syrian conflict remained at several hundred thousand for the first few months of the conflict. A large increase in displacement was first reported in July 2012 when the total number of IDPs was estimated at 1.35 million. Displacement increased again in the later half of 2012 and was estimated at 1.6 million in November 2012. At this time, the largest displaced populations in terms of absolute numbers were in Aleppo, Homs, and Rif Damascus, each of which had an IDP population of between 200,000 and 400,000. In terms of the relative burden of IDPs, these same three governorates also had the greatest proportions displaced at 7 % in Aleppo, 14 % in Homs, and 16 % in Rif Damascus. In total, six governorates had displaced populations exceeding 100,000; IDP populations in Al-Hasakah, Ar-Raqqa, and Idleb were reported as between 100,000 and 200,000.

By February 2013, national IDP estimates increased considerably to 2.4 million and IDP populations exceeded 100,000 in 9 of the 14 governorates; Idleb and Homs had the largest displaced populations, both of which exceed 500,000. With respect to the burden of IDPs relative to the pre-conflict population, the governorates with the largest proportion of displaced included Idleb (42 %), Ar-Raqqa (41 %), Homs (30 %), Deir-ez-Zor (23 %) and Rif Damascus (21 %). In the first half of 2013, increases in the displaced population were exponential and mid-year, national IDP estimates exceeded 5 million. IDP population estimates in this period showed the largest increases in displacement in Rif Damascus and Aleppo, where IDP populations in both governorates exceeded 1 million for the first time. By May 2013, 10 of 14 governorates had IDP populations that exceeded 100,000 and in late 2013, the total number of IDPs was estimated at 6.5 million.

By February 2014, the IDP population reported by ACAPS exceeded 7 million; however, the latest available estimates reported in October 2014 placed the total number of IDPs in Syria back at 6.5 million. With respect to absolute numbers of displaced, governorates with the largest displaced populations in October 2014 included Aleppo (1,787,000), Rif Damascus, (770,000), and Idleb (708,000). In terms of relative burden, described as the proportion of the pre-conflict population that are IDPs, the governorates with the highest burden of displacement were Tartous (47 %) and Rif Damascus (41 %); Dara’a was least affected in terms of both the absolute and proportionate size of the displaced population (Fig. 3). Assessing displaced populations as a proportion of pre-conflict population rather than a crude number may provide a more nuanced understanding of the demographic context in which displacement is occurring. Although Aleppo reported the highest crude number of IDPs in October 2014 (1,787,000), when the displaced population is expressed as percentage of the 2011 pre-conflict population, it has a comparatively low burden of displacement at 30 %. In contrast, Tartous, with a displaced population accounting for 47 % of the pre-conflict population also has an exceptionally high burden of displacement despite the smaller absolute size of the displaced population (452,000).

### Needs assessment

**Fig. 4:**
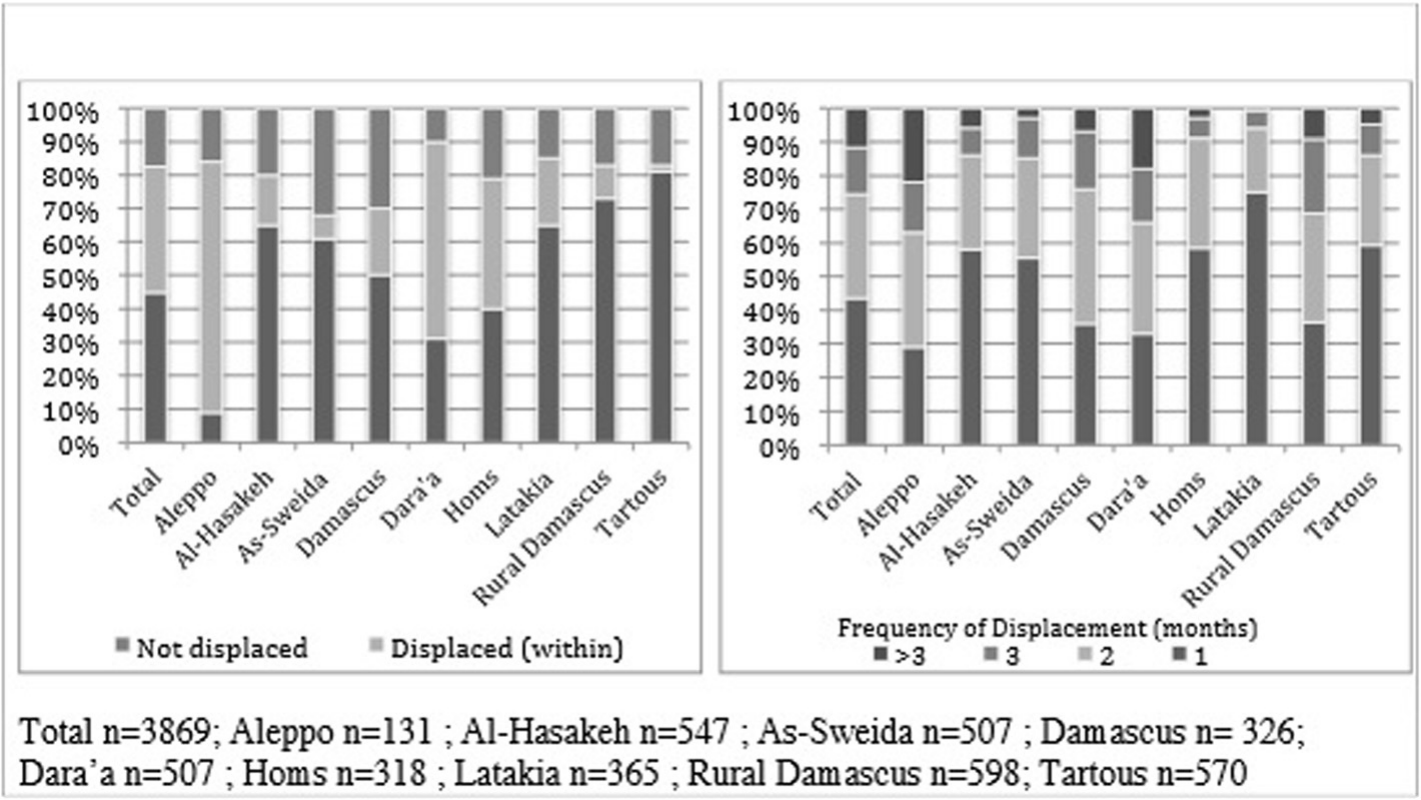
Type and frequency of displacement by governorate among households included in the needs assessment

A majority (82.4 %, CI: 80.7–84.1) of households included in the needs assessment was displaced. Displaced households were categorized into to two groups: those displaced outside their governorate (44.8 %, CI: 42.6–47.0) and those displaced within their governorate (37.7 %, 35.5–39.8); these proportions varied substantially by governorate. Differences in adjusted and unadjusted figures were observed for the variable summarizing displacement from outside or within the governorate; the difference in unadjusted and adjusted proportions is due to the large weight given to Aleppo. Areas with high levels of conflict such as Aleppo, Dara’a, and Homs tended to have larger numbers of households displaced from within the province (Fig. 4). In contrast, IDPs in As-Sweida and Tartous, governorates that have seen lower levels of conflict, were more likely to be displaced from outside the province suggesting that, as anticipated, populations are moving to areas perceived to be more secure. The vast majority of displaced households, 72.3 % (CI: 70.0–74.5) had been displaced for more than a year; 13.7 % (CI: 12.1–15.5) had been displaced for between six months and a year and 13.9 % (CI: 11.7–16.5) for less than six months. The highest proportions of newly displaced households, defined as having been displaced within 3 months preceding the survey, were in Latakia (15.1 %, CI: 11.5–19.6) and As-Sweida (11.3 %, CI: 8.3–15.3). In contrast, the lowest proportions of newly displaced households were found in Aleppo (0.9 %, CI: 0.1–6.3)-) and Damascus (2.8 %, CI: 1.2–6.1)-, presumably because intense fighting in these areas is forcing households to move elsewhere.

Nearly half (43.6 %, CI: 41.2–46.1) of displaced households reported being displaced once; a sizeable proportion (30.9 %, CI: 28.6–33.1) were displaced twice and 25.5 % (CI: 22.4–28.9) were displaced three or more times. The number of times a household was displaced varied by governorate, with households in the highly conflict affected governorates of Dara’a and Aleppo reporting being displaced more times (Fig. 4). Statistically significant differences were also observed between populations displaced within their governorate as compared to those from other governorates frequency of displacement. Households displaced within their governorate were significantly more likely to have moved multiple times as compared to those displaced from other governorates (*p* < 0.001). Nearly half (48.2 %, CI: 44.8–51.5) of households displaced from outside their governorate were displaced one time compared to 38.1 % (CI: 34.6–41.7) of those displaced within their governorate. Only 5.9 % (CI: 4.4–7.6) of households displaced from outside their governorate were displaced three or more times compared to 18.6 % (CI: 15.6–21.6) of those displaced within their governorate.

**Fig. 5:**
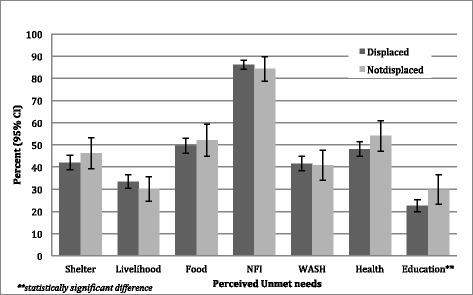
Perceived Priority unmet needs by sector and displacement status

Differences in selected sector-specific indicators were analyzed by displacement status and are presented in Table 3. No significant differences were observed between displaced and non-displaced with respect to living conditions with the exception of crowding where displaced households were significantly less likely to have ≥3 people per sleeping room (37.3 % [CI: 34.0–40.8] vs. 45.4 % [CI: 38.1–52.8], *p* = 0.049). Households that were displaced as compared to non-displaced were significantly more likely to be food insecure (defined as consuming ≤1 meal per day; 12.2 % (CI: 9.7–15.3) vs. 4.8 % (CI: 2.5–9.0), *p* < 0.001); to have household members requiring follow up or specialized medical care (63.4 % [CI: 61.0–65.8] vs. 50.6 % [CI: 45.0–55.8], *p* < 0.001); to be unable to purchase medications (23.0 % [CI: 19.8–26.4] vs. 15.8 % [CI: 11.3–21.6], *p* = 0.031); and to have children not enrolled in school (32.6 % [CI: 30.4–35.0] vs. 24.2 % [CI: 19.9–29.1], *p* = 0.002). Priority unmet needs as perceived by respondents are summarized by sector and displacement status in Fig. 5. A household reporting any specific need within the sector as one of their top five priorities for aid was classified as having an unmet need within that sector. With the exception of education, no significant differences were observed in priority unmet needs between displaced and non-displaced populations. Education was prioritized as unmet need by 29.9 % (CI: 23.2–37.5) of non-displaced households as compared to 22.5 % (CI: 19.8–25.4) of displaced households (*p* = 0.041) despite lower enrollment rates among displaced households.

**Table 3:** Comparison of reported sector-specific indicators by displacement status

	Displaced (n = 2979)			Not Displaced (n = 711)			
	n	%	(95 % CI)	n	%	(95 % CI)	*p*-value
Living conditions							
Shelter is in need of repair	1898	66.8	(62.8–70.6)	417	57.8	(48.1–66.9)	0.075
Poor access to water	1637	26.2	(22.3–30.4)	326	29.8	(13.3–28.4)	0.173
Insufficient toilet access	1637	35.9	(31.5–40.5)	326	32.0	(22.9–42.7)	0.496
3 or more people per sleeping room (crowding)	2753	37.3	(34.0–40.8)	661	45.4	(38.1–52.8)	0.049
Food Security							
Consume ≤1 meal daily	2900	12.2	(9.7–15.3)	679	4.8	(2.5–9.0)	<0.001
Food needs are met by local shops	2799	75.9	(73.6–78.0)	674	78.9	(74.1–83.2)	0.290
Obstacles faced accessing food	2892	90.2	(88.6–91.6)	686	88.4	(84.6–91.8)	0.320
Health access							
Attempted to seek care in last 4 weeks	2742	52.8	(50.3–55.4)	636	58.4	(52.4–63.9)	0.155
Households with members requiring follow up or specialized care	2979	63.4	(61.0–65.8)	732	50.6	(45.0–55.8)	<0.001
Unable to purchase medications in last 4 weeks	2636	23.0	(19.8–26.4)	611	15.8	(11.3–21.6)	0.031
Education							
Household with a school child not enrolled	2979	32.6	(30.4–35.0)	711	24.2	(19.9–29.1)	0.002

## Discussion

The importance of incorporating current and accurate data into humanitarian assistance planning is evident; however, the challenges of effectively enumerating displaced populations often impede such efforts [20]. Attempts to enumerate or estimate IDPs may be clouded by political interests, fundraising, and intra-organizational relationships and often lack continuity and consistency [21]. While approaches have improved over the past decade, few sources of IDP estimates provide detailed methodologies for how they were obtained or clearly acknowledgement conflicts of interest, thereby limiting the ability of the humanitarian community to rigorously appraise them and identify the most reliable estimates. As such, many IDP estimates may be best used to observe trends over time and to complement other available data but not as standalone sources of information.

National level IDP for Syria estimates present five fairly distinct phases of reported conflict-related displacement that coincide with events in Syria: from March 2011 to June 2012, from July 2012 to February 2013, from March to August 2013, from September to February 2014, and from February to October 2014. In March 2011, the peaceful anti-government protests that spread across the nation over several weeks were met with violence from the governing regime. From March 2011 to March 2012, internal displacement was viewed as “temporary and sparse,” characterized by people fleeing conflict hot spots, moving temporarily to surrounding areas or nearby cities, then returning home after protests and violence subsided [22, 23]. By July 2012, the International Committee of the Red Cross and Red Crescent Societies (ICRC) declared that the threshold for an armed civil conflict had been met and displacement, both internal and external, escalated to a new level [24, 25]. The wave of displacement beginning in the second half of 2012 was characterized by the introduction of makeshift IDP camps, first along the Turkish border and later spreading across the country into southern governorates [13].

The scale of displacement continued to increase rapidly in the first half of 2013 with estimates of the displaced population exceeding 4 million by May 2013 [18]. In August 2013, as reports circulated of the use of chemical weapons in the suburbs of Damascus, the crisis entered an even more intensive phase. By September 2013 the Syrian refugee population exceeded 2 million and IDP estimates, while hampered by lack of access due to the deteriorating security situation in many areas, climbed to 6.5 million, with the majority of new displacement occurring in Homs, Idleb, Aleppo, and the northeastern parts of Syria [17]. Mass population movement in northern Syria was seen in late 2013 and early 2014 following continuous aerial bombardment, most notably in eastern Aleppo. In-fighting among opposition forces escalated in 2014 and, though centered in Al-Hasakah and Aleppo, clashes expanded into other governorates causing many that were already displaced to again flee areas previously considered to be safe [14, 18].

Evidence from the needs assessment indicates the displaced population is not highly mobile, with most households reporting being displaced only one (43.6 %, CI: 41.2–46.1) or two times (30.9 %, CI: 28.6–33.1). Areas with high levels of conflict such as Aleppo, Dara’a, and Homs tended to have larger numbers of households displaced from within the governorate. In contrast, IDPs in As-Sweida and Tartous, governorates that have seen lower levels of conflict, were more likely to be displaced from outside the governorate suggesting that, as anticipated, populations are moving to areas perceived to be more secure. The highest proportions of newly displaced households, defined as having been displaced within 3 months preceding the assessment, were in Latakia (15.1 %, CI: 11.5–19.6) and As-Sweida (11.3 %, CI: 8.3–15.3). In contrast, the lowest proportions of newly displaced households were found in Aleppo (0.9 %, CI: 0.1–6.3] and Damascus (2.8 %, CI: 1.2–6.1), presumably because intense fighting in these areas is forcing households to move elsewhere.

Unmet needs were relatively similar between displaced and non-displaced households; however, the ‘non-displaced’ population included in the needs assessment was highly selective and represents an especially vulnerable sub-group of the non-displaced population. Recent conflicts in the region such as those in Iraq and the Gaza Strip provide insight into the toll of sanctions and protracted conflict of this level on civilians [26, 27]. While humanitarian assistance funding is scarce throughout the region, this is only part of the problem. The impact of sanctions, damaged infrastructure, and volatile security concerns on basic service provision for those in Syria is immense, often preventing civilians from accessing assistance, even when supplies are available [27–29]. Attention to long-term planning and reform of existing aid delivery systems, including strengthening local capacity and infrastructure wherever possible, are essential for meeting the needs of IDPs in Syria in the years to come and in the longer-term, for the transition to post-conflict reconstruction [30].

### Limitations

Restricted access by the international community to those inside Syria makes accurate estimation of IDP populations a challenge. Primary IDP population estimates identified in this review draw from formal registration systems established by NGOs, UN agencies, and various organizations providing humanitarian assistance in the country. However, Syria’s division into areas run by the government, those led by the various armed groups, and those areas still contested, makes countrywide monitoring of displacement difficult. Consequently, few primary sources of displacement data are available. As such, the key limitation of the desk review is reliance on one secondary source and few primary sources of data.

Findings from the needs assessment are a strong indication of the widespread unmet needs in Syria among both displaced and non-displaced population. However, they likely under represent the severity of the crisis and the extent of actual humanitarian needs. The limited number interviews conducted in certain highly affected areas, such as Aleppo and Homs, and the inability to access areas not under government control and close to the fighting lines, which are likely to have less access to humanitarian assistance and other basic services is an important limitation of the needs assessment.

## Conclusions

Displacement often corresponds to conflict levels; however, the direction of this relationship is not uniformly supported by governorate-level analysis. A number of governorates reporting a high proportion of the displaced population (Rif Damascus and Aleppo) also have higher levels of conflict mortality and ongoing violence. It is important to note that differences in displacement within governorates may be related to the specific location of conflict and displacement. For example, while displacement and conflict are reportedly high in Homs, the majority of IDPs may be living in more remote areas of the governorate while violence is focused in the city of; as such, the relationship observed between displacement and conflict at the governorate level may not be mirrored on a smaller scale between cities. Governorate level IDP estimates are also influenced by limited access to certain areas, unsubstantiated estimates, and substantial discrepancies in reporting between multiple sources. Figures from some governorates, notably Latakia and Tartous, support the hypothesis that lower levels of conflict are associated with a greater numbers of IDPs as populations are pulled toward safer areas. Conversely, Quneitra and As-Sweida, which also have lower levels of violence and conflict mortality, do not share this burden and host small IDP populations, both in terms of pre-conflict population proportion and absolute numbers.

As the conflict in Syria continues in its fourth year, the frontlines have expanded and security throughout the country, while ever-changing, continues to decline. Violence has expanded in areas previously considered to be safe, leading many families already displaced to relocate multiple times. Secondary displacement is not uniformly reported across sources and it is not always clear how individual IDP figures account for multiple displacements in their estimates. Additional details about displacement, including whether displaced individuals originated within the current governorate or outside of the governorate would further assist in understanding migration trends and humanitarian assistance planning. While levels of unmet need are high in both displaced and non-displaced populations, the scale of conflict affected population and the capacity to provide humanitarian assistance necessitates targeting strategies that includes both displacement and other vulnerability criteria to ensure that the needs of both displaced and non-displaced populations are met. Programming strategies specific to the length of displacement are essential, where newly displaced populations are likely to have very different needs than those displaced in the their current location for an extended period, and will likely vary by location and over time as the conflict evolves.

## Abbreviations

ACAPS: Assessment capacities project
ECHO: European commission humanitarian aid office
IDMC: Internal displacement monitoring centre
IDP: Internally displaced population
ICRC: International committee of the red cross
IS: Islamic State
J-RANS: Joint rapid assessment of Syria
MoLa: Syrian ministry of local administration
OCHA: Organization for the coordination of humanitarian affairs
SARC: Syrian Arab Red Crescent
SINA: Syria integrated needs assessment
SNAP: Syria needs analysis project
UN: United Nations
UNHCR: The United Nations refugee agency
UNICEF: The United Nations children’s fund
